# Supplementary figures and images for: A Gene Signature Identifying CIN3 Regression and Cervical Cancer Survival
Source: Cancers (Basel). 2021 Nov 16;13(22):5737. doi: 10.3390/cancers13225737 (PMC8616457; doi:10.3390/cancers13225737)

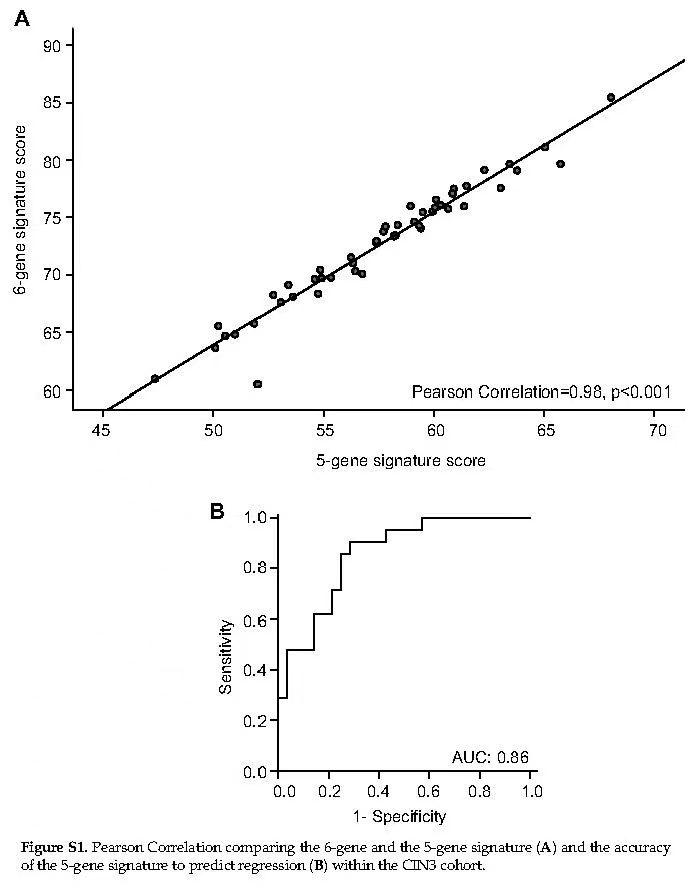

Supplement: Supplementary file 1 [file cancers-13-05737-s001.zip › cancers-1412554-supplementary-figureS1.jpg]
